# Use of brief, simple anxiety assessment tools in palliative care – yes, we can: a cross-sectional observational study of anxiety visual analog scale and numeric rating scale

**DOI:** 10.1186/s12904-025-01814-2

**Published:** 2025-07-01

**Authors:** Adrien Evin, Jean-François Huon, Aurelie Le Thuaut, Patricia Jego, Pierre Nizet, Caroline Victorri-Vigneau, Marianne Bourdon, Palliative care team

**Affiliations:** 1 Nantes Université, Tours Université, CHU Nantes, CHU Tours, INSERM, Methods in patients-centered outcomes and Health Research, SPHERE, Nantes, F-44000 France; 2 Nantes Université, CHU de Nantes, Pharmacie, Nantes, F-44000 France; 3 Nantes Université, CHU de Nantes,Direction de la Recherche et de l’Innovation, Plateforme de Méthodologie et Biostatistique Unit, Nantes, F-44000 France; 4 Nantes Université, CHU de Nantes,Centre d’évaluation et d’information sur la pharmacodépendance-addictovigilance, Nantes, F-44000 France; 5 Integrative Center for Oncology, Angers, Nantes, France; 6 Nantes Université, CHU de Nantes, Service Interdisciplinaire Douleur, Soins Palliatifs et de Support, Médecine intégrative, NANTES , F-44000 France

**Keywords:** Palliative care, Anxiety, Self-report, Numeric rating scale, Visual analog scale, ROC curve, Sensitivity and specificity

## Abstract

**Background:**

Given the high prevalence of anxiety in palliative care and its frequent underestimation by healthcare professionals, it is important to use simplified tools to facilitate the evaluation of anxiety. The State-Trait Anxiety Inventory–State scale is a reference 20-item questionnaire that has been validated in this population, but is too long for some patients. The visual analog scale (VAS) and the numeric rating scale (NRS) are two short instruments that have been validated to assess pain, but not anxiety in palliative care.

**Aim:**

This study sought to investigate the correlation between anxiety VAS and NRS and State-Trait Anxiety Inventory-State scale for assessing anxiety in palliative care patients.

**Methods:**

A single-center cross-sectional observational study was conducted over 2 years. All palliative care patients followed by the palliative care team of a French university hospital were eligible. Each patient completed the State-Trait Anxiety Inventory-State scale and rated their perceived anxiety using the NRS and VAS. Pearson’s correlation test between the scales and receiver operating characteristic (ROC) curve analysis were performed to determine diagnostic cut-offs.

**Results:**

A total of 186 patients were included (89.8% with cancer), 20.4% of whom had severe or very severe anxiety. The NRS/ State-Trait Anxiety Inventory–State scale and VAS/ State-Trait Anxiety Inventory–State scale correlations were 0.62 and 0.70, respectively. The NRS and VAS showed good discrimination, with an area under the ROC curve of 0.81 and 0.88, respectively. Cut-offs of 5 (NRS) and 49 millimeters (VAS) yielded sensitivities of 89.5% and 89.2%, respectively, for the detection of severe or very severe anxiety, with specificities of 60.1% and 70.3%, respectively.

**Conclusion:**

Cut-offs of 5 for the NRS and 49 millimeters for the VAS showed excellent sensitivity for detecting anxiety in palliative care.

**Supplementary Information:**

The online version contains supplementary material available at 10.1186/s12904-025-01814-2.

## Introduction

Anxiety and worry are often part of the end-of-life experience [1]. With the increasing prevalence of deaths from chronic rather than acute diseases [2, 3], many patients endure the unpredictability and long-term nature of chronic illness for several years [4]. As a result, there is a growing population that experiences anxiety in palliative situations and over a prolonged period of time in relation to serious and incurable illness. Patient anxiety is an issue that deserves particular attention from healthcare professionals working in palliative care, as confirmed by the prevalence reported in studies. For instance, the prevalence of anxiety in patients with advanced cancer, acquired immunodeficiency syndrome, heart disease, chronic obstructive pulmonary disease, and renal disease ranges from 13 to 74% [3].More recent studies have reported similar prevalence rates, among patients with advanced cancer—the population for which the most robust data are available—prevalence rates range from 26 to 61% [1, 5–9]. This anxiety has multiple detrimental consequences: it increases the perceived burden of illness, negatively affects quality of life, and raises the likelihood of hospitalization [6, 9–11]. Patient anxiety is therefore a critical concern that requires focused attention from healthcare professionals.

Anxiety has traditionally been divided into state anxiety and trait anxiety [12, 13]. State anxiety refers to the experience of anxiety at the time of completion and is relevant to assessing stress in a specific situation [14], unlike trait anxiety, which is related to a personality trait. This symptom is regularly underestimated by health professionals, who emphasize the value of self-report scales [15]. The State-Trait Anxiety Inventory [14, 16], a commonly used self-report measure of anxiety, distinguishes between state and trait anxiety with two scales. Each scale consists of 20 items, so that the State-Trait Anxiety Inventory–State scale provides a detailed understanding of the state of anxiety. However, completing 20 items is a cognitive task that may sometimes be inappropriate for patients who are often described as polysymptomatic [17, 18].

It seems important to develop less cognitively demanding tools to facilitate the assessment and support of anxiety in palliative care patients, such as a visual analog scale (VAS) or a numeric rating scale (NRS), which are validated tools for assessing pain [19]. Using tool formats that clinicians are already familiar with to assess other symptoms may facilitate their integration into clinical practice, especially since their brevity and simplicity make them suitable for patients who are often physically fatigued. These simplified tools, consisting of only one short question, have been developed to facilitate the assessment and follow-up of pain. While anxiety VAS and NRS have been used to assess anxiety in anesthesia and dental care [20, 21], to the best of our knowledge, they have never been studied in palliative care [22]. In this population, the Edmonton Symptom Assessment System [23] is a method for assessing symptoms using a VAS or NRS [24]. Unlike short anxiety scales (such as anxiety VAS or NRS), this tool assesses at least eight different symptoms within the same rating scale. The item ‘feeling nervous’ is commonly used to assess anxiety. This tool has not been further validated, as the authors of a review point out [25]: “for many symptoms, there is no clear evidence of optimal thresholds”, with only three studies examining the symptom of anxiety and none performing a comparison with a reference symptom intensity scale such as the State-Trait Anxiety Inventory-State scale. A recent article [26] highlights the challenge of finding an appropriate cut-off for this instrument. Therefore, there is still a need to develop tools adapted to the assessment of anxiety [27].

It would be helpful to determine whether the information provided by the anxiety VAS and NRS is reliable for assessing state anxiety in palliative care patients. This would allow researchers and clinicians to draw on these scores when the State-Trait Anxiety Inventory-State scale is not applicable to patients. The use of short, validated scales can be an effective first-line screening method to help identify people who may need further psychological assessment, as recommended in the latest cancer guidelines, for example.

This study thus aimed to investigate the correlation between anxiety scores on the VAS and NRS scales and the State-Trait Anxiety Inventory-State scale for assessing anxiety in palliative care patients. Besides, the discriminative ability of the anxiety VAS and NRS and their optimal cut-offs were explored.

## Methods

### Study design

A single-center cross-sectional observational study was conducted over a 2-year period (March 2018 to March 2020) in a French university hospital. Each patient completed the State-Trait Anxiety Inventory-State scale and rated their perceived anxiety using a NRS and a VAS.

### Population

Adult palliative care patients followed by the mobile palliative care team or hospitalized in the palliative care unit of a French university hospital center were eligible for inclusion. Patients were not eligible if they were unable to complete the questionnaires, had cognitive impairment, were under any form of legal guardianship, or were not fluent in French.

They were enrolled at the first meeting with the palliative care team after the start of the study (which was not necessarily the first time they were seen by a palliative care team). A palliative care physician was responsible for enrollment and questionnaire distribution.

### Measurements and data collection

Demographic and medical data were either reported by the patients or collected from their medical records. These included: sex, age, primary pathology (the primary condition for which the patient was being followed up), and patient follow-up status (by the mobile team or hospitalized in the palliative care unit). All current treatments were listed. The number of different medications patients were taking at the time of the anxiety assessment was reported, including anxiolytic treatments (such as benzodiazepines, Z-drugs, buspirone, antidepressants, antihistamines, and antipsychotics).

State anxiety (i.e., anxiety at the time of completion) was assessed using three self-administered questionnaires (paper form) at the time of patient inclusion in the study:

1. An anxiety VAS (Supplementary Material 1). It consisted of a 100 millimeters horizontal line anchored on the left side by the words “no anxiety” with a happy smiley face and on the right side by “worst possible anxiety” with a sad smiley face. The instruction was as follows: “Please place a vertical mark on the line corresponding to the intensity of anxiety felt.’’ The intensity of anxiety felt was then expressed in millimeters from 0 to 100 millimeters (mm).
2. An anxiety NRS (Supplementary Material 2). Patients answered the following question: “Give the number corresponding to your anxiety between 0 (no anxiety) and 10 (maximum anxiety imaginable)”. The intensity of anxiety felt could vary from 0 to 10.
3. The State-Trait Anxiety Inventory-State scale (Supplementary Material 3). This tool has been validated in French and is used in cancer and non-cancer pathologies [14, 16]. It consists of 20 items, each rated on a 4-point scale. The total score can range from 20 to 80. A higher score indicates greater anxiety. The calculation of the total score tolerates a maximum of two missing data. Missing data are then imputed by averaging the responses to the other items. According to the manual, results can be interpreted as follows: “very high state of anxiety” >65, “high” from 56 to 65, “moderate” from 46 to 55, “low” from 36 to 45, and “very low” ≤35.

### Statistical analyses

Patient characteristics at the time of inclusion were described using the number and percentage of each modality for qualitative variables and the mean, standard deviation (SD), minimum, and maximum for quantitative variables.

We examined the criterion validity (“that refers to the extent to which scores on a particular instrument relate to a gold standard” [28]) by testing the correlation between the anxiety VAS, the anxiety NRS, and State-Trait Anxiety Inventory-State scale (our gold standard). Pearson’s correlations were measured between NRS/State-Trait Anxiety Inventory-State scale and VAS/State-Trait Anxiety Inventory-State scale were measured. The correlation was considered weak for < 0.4, moderate between 0.4 and 0.69, and strong for ≥ 0.7 [29]. This procedure is necessary to validate the quality of a measurement tool [28].

Receiver operating characteristic (ROC) curve analyses were performed to determine the discrimination of the tools and the best cut-offs for diagnosing severe or very severe anxiety (defined as State-Trait Anxiety Inventory-State scale score ≥ 56). The ROC curve illustrates the trade-off between sensitivity and 1 minus specificity across various possible thresholds used to classify a test result as positive. Sensitivity and specificity were calculated (with 95% confidence intervals [CI]) to identify the most appropriate cut-off. In this context, sensitivity reflects the ability of the test to correctly identify individuals with high anxiety according to the State-Trait Anxiety Inventory-State scale (score ≥ 56), while specificity reflects the ability to correctly identify individuals without high anxiety based on the same gold standard [30]. Explanations of the methods used to calculate the different elements can be found in Supplementary Material 4. For discrimination, the area under the curve (AUC) was calculated (with 95% CIs). Discrimination was considered poor for AUC < 0.60, possibly useful between 0.6 and 0.75, and clearly useful for > 0.75 [31].

Data were analyzed using SAS software v9.4 (Cary, NC, USA).

### Ethical considerations and patient information

Each patient received oral and written information about the study. This study was conducted in accordance with French regulations. Patients gave oral informed consent in accordance with current French regulations. This project was approved by the hospital’s local ethics committee “Groupe Nantais d’Ethique dans le Domaine de la Santé” (GNEDS) on April 03, 2018.

## Results

### Population characteristics

A total of 186 patients were enrolled in the study, 58.1% of whom were male. The mean age was 66.4 years (min 22–max 96) and the most common pathology was cancer (Table 1). Of the participants, 66.7% were enrolled during their hospitalization in the palliative care unit, and 60.8% were meeting the palliative care team for the first time. For the remaining 73 patients, the median number of previous appointments with a palliative care team was 2 (Q1-Q3 [1.00;3.00]). The average number of medications per patient was 8.4 (min 0–max 23), including 68.3% of patients with current anxiolytic treatment (benzodiazepines, Z-drugs, buspirone, antidepressants, antihistamines, and antipsychotics). 49.5% of patients were receiving benzodiazepines.

**Table 1:** Description of the population

		Total *N* = 186
Sex		
	Men (%)	108 (58.1%)
	Women (%)	78 (41.9%)
Age (years)		
	Mean (SD)	66.42 (13.7)
	Min-max	[22.0;96.0]
Main pathology		
	Cancer (%)	167 (89.8%)
	Organ failure (%)	11 (5.9%)
	Polypathology (%)	7 (3.8%)
	Neurodegenerative disease (%)	1 (0.5%)
Care team		
	Mobile palliative care team (%)	62 (33.3%)
	Palliative care unit (%)	124 (66.7%)

SD: standard deviation

### SD: standard deviation

The mean State-Trait Anxiety Inventory-State scale score was 42.6 (min 20–max 75; SD 14.4), with 20.4% (95% CI: 12.6–25.4%) of patients having high or very high anxiety (Table 2).

**Table 2:** Description of the State-Trait anxiety Inventory-State score

State-Trait Anxiety Inventory-State score	Total *N* = 186
Very high anxiety (≥ 65)	17 (9.1%)
High (56–65)	21 (11.3%)
Average (46–55)	35 (18.8%)
Low (36–45)	46 (24.8%)
Very low (≤ 35)	67 (36.0%)

For the two short scales, the mean NRS score was 4.2 (min 0.0–max 10.0; SD 2.5) and the mean VAS score was 39.8 mm (min 0.0–max 100.0; SD 25.7).

### Criterion validity

The correlation was moderate for NRS/State-Trait Anxiety Inventory-State scale, with a Pearson correlation coefficient (r) of 0.62, and strong for VAS/State-Trait Anxiety Inventory-State scale, with *r* = 0.70. The correlation was strong for NRS/VAS, with *r* = 0.89.

### Discrimination and cut-off choice

**Fig. 1:**
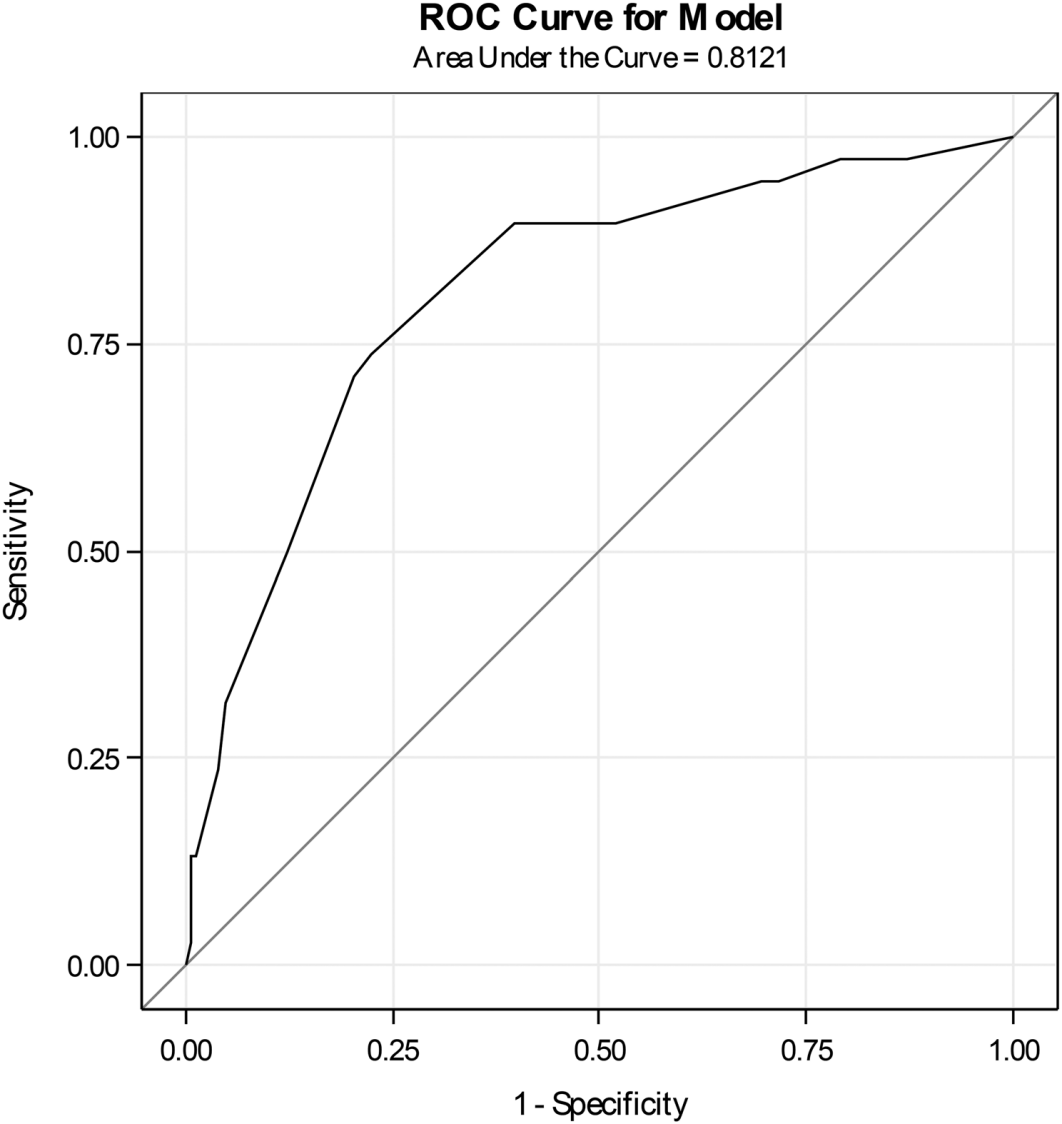
Receiver operating characteristic (ROC) curve of anxiety NRS

**Fig. 2:**
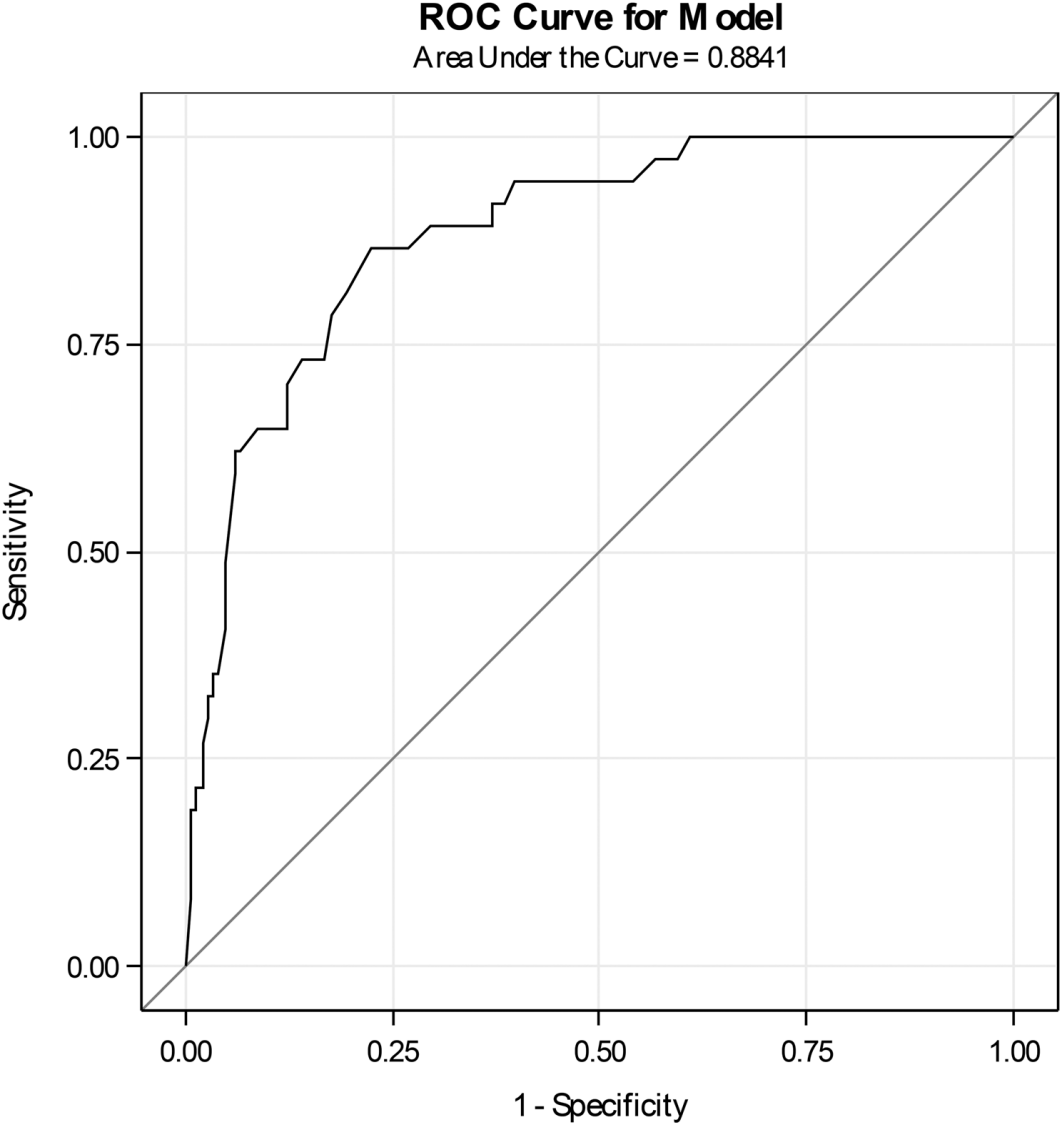
Receiver operating characteristic (ROC) curve of anxiety VAS

Discrimination of the NRS and the VAS was good, with an area under the ROC curve of 0.81 (95% CI: 0.73–0.89) (Fig. 1) for the NRS and 0.88 (95%CI: 0.83–0.94) (Fig. 2) for the VAS.

A cut-off of 5 for the NRS yielded a sensitivity of 89.5% (95% CI: 75.2–97.1) for detecting severe or very severe anxiety, with a specificity of 60.1% (95% CI: 51.8–68.1), according to ROC curve analyses.

A cut-off of 49 mm for the VAS yielded a sensitivity of 89.2% (95% CI: 74.6–97.0), with a specificity of 70.3% (95% CI: 62.2–77.5). These cut-offs offered the best compromise between sensitivity and specificity.

## Discussion

This study showed that the two short anxiety assessment tools (anxiety NRS and VAS) correlated well with the reference scale (State-Trait Anxiety Inventory-State scale), with good discrimination (AUC > 0.8). Such simple, short, and easy-to-use tools, similar to those used for pain assessment, are of interest both clinically to assess and monitor this symptom more rapidly, and for research, as the multiplicity of questionnaires is often a barrier in these populations. In our single-center observational study, 20.4% (95% CI: 12.6–25.4) of 186 patients reported high or very high anxiety (State-Trait Anxiety Inventory-State scale). We also established cut-offs for the two brief scales to identify patients with severe or very severe anxiety. Cut-offs of ≥ 5 for the NRS and ≥ 49 mm for the VAS demonstrated high sensitivity along with acceptable specificity levels to limit the risk of overdiagnosis, making them suitable for screening purposes. These thresholds serve as clinically useful indicators at which healthcare professionals can suspect elevated anxiety levels. Although less precise than the State-Trait Anxiety Inventory-State scale, these brief instruments are easier to administer and can facilitate initial discussions with patients about their psychological state and potential need for further support—an approach that is often sufficient in routine clinical practice.

### Prevalence of anxiety and polypharmacy in palliative care patients

In this study, 20.4% of patients reported high or very high anxiety (State-Trait Anxiety Inventory-State scale), highlighting that our population had a high level of anxiety. This rate increases to 39.3% when patients with moderate anxiety are included. Taking into account the methodological limitations due to the variety of assessment tools and symptom screening cut-offs, the prevalence in our sample seems to be in line with previous studies (13–79%) [3]. Despite the high prevalence of anxiety as a symptom in palliative care patients, as shown in our study, routine assessment of anxiety remains low compared to pain assessment [32].

The prevalence of polypharmacy is evident in our study, with an average number of 8.4 treatments per patient. These findings are consistent with recent literature reviews on polypharmacy in palliative care [33]. In our sample, 68.3% of patients were receiving anxiolytic treatment at the time of assessment, which may have contributed to the lower observed prevalence of anxiety. The high rate of benzodiazepine use is consistent with previous studies reporting prevalence rates of 58% and 74% [34, 35]. Benzodiazepines are considered by many palliative care physicians to be one of the four essential drugs [36]; however, recent literature highlights concerns about their potential overuse and misuse [37, 38]. Given that the rate of benzodiazepine prescription exceeds the actual prevalence of anxiety symptoms, the appropriateness of their use warrants critical evaluation. Further research is needed to better understand the relationship between anxiety prevalence and the proportion of patients receiving anxiolytic therapy. Additionally, the role of non-pharmacological approaches and psychological support should be explored as part of a comprehensive anxiety management strategy. There is also a need for tools to assist in the appropriate use of medications, particularly in the context of deprescribing. Monitoring of symptom evolution [39] appears to be a valuable tool for assessing the efficacy of therapeutic adjustments. In addition, the use of anxiety NRS and VAS may prove useful in monitoring the evolution of this symptom.

### Correlation with reference scale and cut-off selection

Our results for these two scales, whether in terms of correlation or cut-off choice, appear to be consistent with the literature (Table 3). A literature review found over 70 articles on anxiety VAS, but none related to the palliative care population [22].

**Table 3:** Summary and comparison with our results from studies evaluating the correlation between short anxiety rating scales and the State-Trait anxiety Inventory-State scale

Study (author, year)	Population	Brief scale used	Correlation with STAI-S	STAI-S cut-off	Proposed cut-off for brief scale used	Sensitivity / specificity
Our results	Palliative care patients (*n* = 186)	VAS NRS	*r* = 0.70 *r* = 0.62	56	5/10 49/100	Se: 89.2% / Sp: 60.1% Se 89.5% / Sp 70.3%
Lavedán Santamaría et al., 2022	Nursing students (*n* = 185)	VAS	*r* = 0.686	30	> 60/100	Se: 73% / Sp: 77%
Ducoulombier et al., 2020	Hospitalized patients with pain (*n* = 392)	VAS	*r* = 0.67 (95% CI: 0.61–0.72)	45	≥ 40/100	Se: 81% / Sp: 70%
Labaste et al., 2019	Post-anesthesia patients (*n* = 500)	VAS	*r* = 0.555 at admission; *r* = 0.593 at discharge	40	> 34/100	Se: 81.6% / Sp: 80%
Hepp et al., 2021	Women undergoing caesarean section (*n* = 47)	VAS	*r* = 0.76 (admission); *r* = 0.60 (closure); *r* = 0.65 (2 h post-op)	Not studied	Not studied	Not studied
Facco et al., 2013	Pre-anesthesia patients (*n* = 100)	VAS	*r* = 0.50	40	46/100	Se: 83% / Sp: 61%
Davey et al., 2007	Women attending breast clinic (*n* = 487)	VAS	*r* = 0.78 (95% CI: 0.73–0.82)	Not studied	Not studied	Not studied
Kindler et al., 2000	Preoperative patients (*n* = 486)	VAS (anesthesia-related fear)	*r* = 0.55	45	Multiple cut-offs tested, none retained e-g- 40/100	Se: 56.2% / Sp: 81%
Luyk et al., 1988	Pre-dental anesthesia (*n* = 45)	VAS	*r* = 0.76	Not studied	Not studied	Not studied
Abend et al., 2014	Undergraduate students (*n* = 172)	VAS	*r* = 0.60	Not studied	Not studied	Not studied
Prokopowicz et al., 2022	Postpartum women (*n* = 200)	NRS	*r* = 0.807 (T1); *r* = 0.778 (T2)	40	≥ 3.5/10	Se: 80%, Sp: 84–85%

NRS: numeric rating scale; Se: sensitivity; Sp: specificity; STAI-S = State-Trait Anxiety Inventory – State scale; VAS: visual analog scale

The data in this article suggest that it is important to study VAS in the population for which it is intended, given the wide disparity in results between different populations. Correlation coefficients between scales vary between studies, e.g. *r* = 0.78 for women followed in a breast clinic [40], *r* = 0.76 for dental anxiety [20], *r* = 0.67 for patients with pain, and, less, *r* = 0.55 for postoperative anxiety [41]. For the same population at different time points, this correlation may change, as highlighted by the study of women undergoing caesarean section. [42], with a correlation coefficient ranging from 0.76 to 0.65. Cut-offs vary widely from study to study. For example, cut-offs in the adult population (for preoperative anxiety) are around 46 mm [21], but with the State-Trait Anxiety Inventory-State scale cut-off at 40, or around 40 mm with the state-Trait Anxiety Inventory-State scale cut-off at 45 for pain patients [43]. The cut-off we chose to distinguish anxious from non-anxious patients using the State-Trait Anxiety Inventory-State scale was 56. In contrast to most studies [21, 41], we chose to consider the intensity of the symptom and not just its presence. The anxiety VAS has also been compared with the State-Trait Anxiety Inventory-State scale in nursing students [44], with a positive correlation (*r* = 0.686) found between the VAS and the State-Trait Anxiety Inventory-State scale. Thus, we could have a common tool for both patients and healthcare workers, which could facilitate its daily use. This scale has even been studied in computerized form to facilitate its use either in research or in the patient’s home for follow-up, with interesting results [45].

The anxiety NRS has been less studied than the anxiety VAS. This scale has recently been studied in the postpartum period [46]. The correlations between State-Trait Anxiety Inventory-State scale and the NRS were high (*r* = 0.807), and at a cut-off of 3.5 (State-Trait Anxiety Inventory-State scale cut-off of 40), the sensitivity was 0.803 and the specificity was 0.843. We chose cut-offs with high sensitivity and lower specificity to detect the presence of an anxiety symptom of significant intensity, to allow for rapid treatment adjustment and more detailed symptom assessment.

The use of cut-offs seems to be controversial; in fact, some studies [47] tend to show that the threshold depends on the patient and it would be undesirable to generalize it. We believe that the existence of a cut-off may be useful for health professionals to facilitate the detection of a symptom.

### Strengths and weaknesses

This study has several strengths that contribute to its significance. We studied two different scales simultaneously and compared them with a reference scale. The number of patients is another strength of our study for this type of population, and these patients were either inpatients or outpatients, thus diversifying the sample. Approximately 90% of our patients had cancer, which may call into question the use of these tools in other pathology categories, but we were still able to include 10% of other pathologies in our study. This overrepresentation of cancer in palliative care remains a reality in palliative care today. The diversity of our sample is a strength of our study, which did not focus solely on cancer patients. These elements increase the generalizability of the results. The study committee was interprofessional and included physicians, pharmacists, and psychologists, providing a multidisciplinary perspective on methodology and analysis. Finally, from an ethical point of view, each patient could benefit from psychological follow-up if a need for support was expressed during the course of the protocol.

However, certain limitations inherent to the study design must be acknowledged. As a single-center and single-country study, this could potentially introduce bias. It was difficult to determine the exact number of people eligible for the study, not least because of the variable nature of the mobile palliative care team interventions. We could be accused of selection bias, but our data, whether in terms of anxiety prevalence or correlation, are close to the literature. The choice of the State-Trait Anxiety Inventory-State scale as the reference scale may be debated, but this scale has very good psychometric data; there is no recommendation based on robust data to favor one scale over another [27, 46, 48, 49]. As with many studies of this type, we did not calculate the sample size. This limitation is common to many studies of this kind, and in our case the calculation was complex because we did not have prevalence data with the State-Trait Anxiety Inventory-State scale in our population, and prevalence was highly variable in the literature [50]. Regarding the data, it would also have been interesting to collect the presence of non-pharmacological treatments in addition to anxiolytic treatments. Finally, we did not examine the psychometric properties of these scales, such as reliability and responsiveness, and this remains to be done in the coming years.

## Conclusion

Despite the high prevalence of anxiety in palliative care patients, this symptom remains underassessed in daily clinical practice, especially when compared to pain. Our study showed that the anxiety NRS and VAS, two simple and rapid tools, correlate well with the reference scale and may serve as effective first-line instruments to screen for severe or very severe anxiety. We proposed cut-offs of 5 for the NRS and 49 mm for the VAS to identify patients likely to experience significant anxiety. These are screening tools, not diagnostic tools, and they should be followed by more specific evaluation if scores are elevated. Nevertheless, they are a practical means of improving the early detection of anxiety and aiding therapeutic decision-making, including symptom monitoring over time. These tools are simple, brief, and easy to use—similar to those used in pain assessment—and are particularly valuable in both clinical and research contexts. Clinically, they allow for more rapid identification and follow-up of anxiety symptoms. From a research perspective, their brevity reduces the burden of multiple questionnaires, which can be a significant barrier in this population. Further studies are needed to validate these tools across more diverse pathologies and to better understand their psychometric properties, particularly their sensitivity to change, which is critical for monitoring the effects of therapeutic interventions.

## Electronic supplementary material

Below is the link to the electronic supplementary material.

Supplementary Material 1

Supplementary Material 2

Supplementary Material 3

Supplementary Material 4

## Data Availability

Data are available upon reasonable request.

## Abbreviations

AUC: Area under the curve
CI: Confidence interval
NRS: Numeric rating scale
ROC: Receiver operating characteristic
SD: Standard deviation
STAI-S: State-Trait Anxiety Inventory–State scale
VAS: visual analogue scale
