# Supplementary material for: Use of brief, simple anxiety assessment tools in palliative care – yes, we can: a cross-sectional observational study of anxiety visual analog scale and numeric rating scale
Source: BMC Palliat Care. 2025 Jul 1;24:173. doi: 10.1186/s12904-025-01814-2 (PMC12211726; doi:10.1186/s12904-025-01814-2)
Supplement: Supplementary file 1 — Supplementary Material 1 [file 12904_2025_1814_MOESM1_ESM.docx]

**Supplementary material 2:** Anxiety- Numerical Rating Scale (French and English version)

**ECHELLE NUMERIQUE DE L’ANXIETE (EN)**

Donner le chiffre correspondant à votre évaluation

De votre anxiété entre 0 (aucune anxiété) et 10 (anxiété maximale imaginable)

**I____I I____I , I____I**

**ANXIETY- Numerical Rating Scale (NRS)**

Please enter the number corresponding to your assessment

Anxiety between 0 (no anxiety) and 10 (maximum anxiety imaginable)

**I____I I____I , I____I**
