# Supplementary material for: Use of brief, simple anxiety assessment tools in palliative care – yes, we can: a cross-sectional observational study of anxiety visual analog scale and numeric rating scale
Source: BMC Palliat Care. 2025 Jul 1;24:173. doi: 10.1186/s12904-025-01814-2 (PMC12211726; doi:10.1186/s12904-025-01814-2)
Supplement: Supplementary file 2 — Supplementary Material 2 [file 12904_2025_1814_MOESM2_ESM.docx]

**Supplementary material 3:** State-Trait Anxiety Inventory-State (French version)

**INVENTAIRE D’ANXIETE ETAT (STAI-Y A)**

*Un certain nombre de phrases que l’on utilise pour se décrire sont données ci-dessous.*

*Lisez chaque phrase, puis entourez, parmi les 4 points à droite, celui qui correspond le mieux à ce que vous ressentez* ***A L’INSTANT****,* ***JUSTE EN CE MOMENT****.*

*Il n’y a pas de bonnes ni de mauvaises réponses. Ne passez pas trop de temps sur l’une ou l’autre de ces propositions et indiquez la réponse qui décrit le mieux vos sentiments actuels.*

|  | Non | Plutôt non | Plutôt oui | Oui |
| --- | --- | --- | --- | --- |
| 1. Je me sens calme | **.** | **.** | **.** | **.** |
| 1. Je me sens en sécurité, sans inquiétudes, en sûreté | **.** | **.** | **.** | **.** |
| 1. Je suis tendu(e), crispé(e) | **.** | **.** | **.** | **.** |
| 1. Je me sens surmené(e) | **.** | **.** | **.** | **.** |
| 1. Je me sens tranquille, bien dans ma peau | **.** | **.** | **.** | **.** |
| 1. Je me sens ému(e), bouleversé(e), contrarié(e) | **.** | **.** | **.** | **.** |
| 1. L’idée de malheurs éventuels me tracasse en ce moment | **.** | **.** | **.** | **.** |
| 1. Je me sens content(e) | **.** | **.** | **.** | **.** |
| 1. Je me sens effrayé(e) | **.** | **.** | **.** | **.** |
| 1. Je me sens à mon aise (je me sens bien) | **.** | **.** | **.** | **.** |
| 1. Je sens que j’ai confiance en moi | **.** | **.** | **.** | **.** |
| 1. Je me sens nerveux (nerveuse), irritable | **.** | **.** | **.** | **.** |
| 1. J’ai la frousse, la trouille (j’ai peur) | **.** | **.** | **.** | **.** |
| 1. Je me sens indécis(e) | **.** | **.** | **.** | **.** |
| 1. Je suis décontracté(e), détendu(e) | **.** | **.** | **.** | **.** |
| 1. Je suis satisfait(e) | **.** | **.** | **.** | **.** |
| 1. Je suis inquiet (inquiète), soucieux (soucieuse) | **.** | **.** | **.** | **.** |
| 1. Je ne sais plus où j’en suis, je me sens déconcerté(e), dérouté(e) | **.** | **.** | **.** | **.** |
| 1. Je me sens solide, posé(e), pondéré(e), réfléchi(e) | **.** | **.** | **.** | **.** |
| 1. Je me sens de bonne humeur, aimable | **.** | **.** | **.** | **.** |
