# Supplementary material for: Use of brief, simple anxiety assessment tools in palliative care – yes, we can: a cross-sectional observational study of anxiety visual analog scale and numeric rating scale
Source: BMC Palliat Care. 2025 Jul 1;24:173. doi: 10.1186/s12904-025-01814-2 (PMC12211726; doi:10.1186/s12904-025-01814-2)
Supplement: Supplementary file 3 — Supplementary Material 3 [file 12904_2025_1814_MOESM3_ESM.docx]

**Supplementary material 4**

**Definitions:**

**True Positives (TP)**: People with high anxiety on both the State-Trait Anxiety Inventory-State scale and the short scale.

**False Negatives (FN)**: People with high anxiety on the State-Trait Anxiety Inventory-State scale, but not detected by the short scale.

**True Negatives (TN)**: People with low anxiety on both tests.

**False Positives (FP)**: People not anxious on the State-Trait Anxiety Inventory-State scale but wrongly identified as anxious by the short scale.

**The methods used to calculate:**

**Sensitivity** = TP / (TP + FN)

**Specificity** = TN / (TN + FP)
