# Supplementary material for: Use of brief, simple anxiety assessment tools in palliative care – yes, we can: a cross-sectional observational study of anxiety visual analog scale and numeric rating scale
Source: BMC Palliat Care. 2025 Jul 1;24:173. doi: 10.1186/s12904-025-01814-2 (PMC12211726; doi:10.1186/s12904-025-01814-2)
Supplement: Supplementary file 4 — Supplementary Material 4 [file 12904_2025_1814_MOESM4_ESM.docx]

**Supplementary material 1:** Anxiety- Visual Analogue Scale (French and English version)

**ECHELLE VISUELLE ANALOGIQUE DE L’ANXIETE (EVA)**

Tracer sur la ligne un trait vertical correspondant à l’intensité ressentie


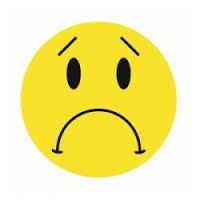

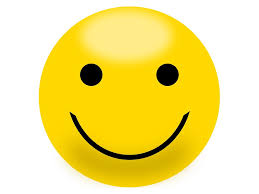


Anxiété

maximale imaginable

Aucune anxiété

**ANXIETY- VISUAL ANALOGUE SCALE (VAS)**

Please place a vertical mark on the line corresponding to the intensity of anxiety felt.


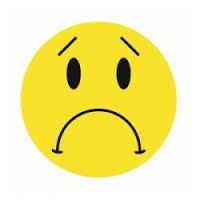

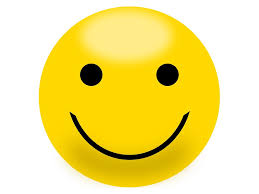


Maximum anxiety imaginable

No anxiety
